# Introgression of the *Aegilops speltoides Su1-Ph1* Suppressor into Wheat

**DOI:** 10.3389/fpls.2017.02163

**Published:** 2017-12-20

**Authors:** Hao Li, Karin R. Deal, Ming-Cheng Luo, Wanquan Ji, Assaf Distelfeld, Jan Dvorak

**Affiliations:** ^1^Department of Plant Sciences, University of California, Davis, Davis, CA, United States; ^2^College of Agronomy, Northwest A&F University, Yangling, China; ^3^School of Plant Sciences and Food Security, Tel Aviv University, Tel Aviv, Israel

**Keywords:** homoeologous chromosome pairing, *in situ* hybridization, MAS, *Ph1*, *ZIP4*

## Abstract

Meiotic pairing between homoeologous chromosomes in polyploid wheat is inhibited by the *Ph1* locus on the long arm of chromosome 5 in the B genome. *Aegilops speltoides* (genomes SS), the closest relative of the progenitor of the wheat B genome, is polymorphic for genetic suppression of *Ph1.* Using this polymorphism, two major suppressor loci, *Su1-Ph1* and *Su2-Ph1*, have been mapped in *Ae. speltoides. Su1-Ph1* is located in the distal, high-recombination region of the long arm of the *Ae. speltoides* chromosome 3S. Its location and tight linkage to marker *Xpsr1205-3S* makes *Su1-Ph1* a suitable target for introgression into wheat. Here, *Xpsr1205-3S* was introgressed into hexaploid bread wheat cv. Chinese Spring (CS) and from there into tetraploid durum wheat cv. Langdon (LDN). Sequential fluorescence *in situ* hybridization and genomic *in situ* hybridization showed that an *Ae. speltoides* segment with *Xpsr1205-3S* replaced the distal end of the long arm of chromosome 3A. In the CS genetic background, the chromosome induced homoeologous chromosome pairing in interspecific hybrids with *Ae. peregrina* but not in progenies from crosses involving alien disomic substitution lines. In the LDN genetic background, the chromosome induced homoeologous chromosome pairing in both interspecific hybrids and progenies from crosses involving alien disomic substitution lines. We conclude that the recombined chromosome harbors *Su1-Ph1* but its expression requires expression of complementary gene that is present in LDN but absent in CS. We suggest that it is unlikely that *Su1-Ph1* and *ZIP4-1*, a paralog of *Ph1* located on wheat chromosomes 3A and 3B and *Ae. tauschii* chromosome 3D, are equivalent. The utility of *Su1-Ph1* for induction of recombination between homoeologous chromosomes in wheat is illustrated.

## Introduction

In most allopolyploid plants, only homologous chromosomes pair in meiosis and only bivalents are present at metaphase I (MI); pairing between homoeologous chromosomes (heterogenetic chromosome pairing) is excluded (Jenczewski and Alix, 2004). The best-known example of genetic exclusion of heterogenetic chromosome pairing is in tetraploid and hexaploid wheat (Okamoto, 1957; Riley and Chapman, 1958; Sears and Okamoto, 1958).

The chromosome complement of tetraploid wheat (*Triticum turgidum* L., 2*n* = 4*x* = 28) consists of the A genome, which was contributed by *T. urartu* Thum., and the B genome, which was contributed by an extinct or undiscovered species closely related to *Aegilops speltoides* Tausch (genomes SS≈BB) (Dvorak and Zhang, 1990; Dvorak et al., 1993). The chromosome complement of hexaploid wheat (*T. aestivum* L., 2*n* = 6*x* = 42) consists of the A and B genomes of tetraploid wheat and the D genome, which was contributed by *Ae. tauschii* Coss. (Kihara, 1944; McFadden and Sears, 1946; Wang et al., 2013). While the chromosomes of these diploid species extensively pair in hybrids among them, virtually no chromosome pairing take places in haploids derived from polyploid wheat (Kimber and Riley, 1963; McGuire and Dvořák, 1982; Jauhar et al., 1991, 1999). This paradox is caused by the expression of the *Ph1* locus on chromosome 5B of tetraploid and hexaploid wheat, which prevents pairing between homoeologous chromosomes (Okamoto, 1957; Riley and Chapman, 1958; Sears and Okamoto, 1958). If *Ph1* is absent due to aneuploidy (Riley, 1960) or a deletion, such as *ph1b* in hexaploid wheat (Sears, 1977) and *ph1c* in tetraploid wheat (Giorgi, 1978), meiotic pairing of homoeologous chromosomes is restored.

The *Ph1* locus on chromosome 5B was initially associated with a cluster of cyclin-dependent kinase 2 (*Cdk2*)-like protein genes including a DNA fragment translocated from chromosome 3A (Griffiths et al., 2006). The translocated fragment was shown to contain *TaZIP4-B2*, a highly expressed paralog of *TaZIP4-1*, which is located on wheat chromosomes 3A (*TaZIP4-A1*), 3B (*TaZIP4-B1*), and 3D (*TaZIP4-D1*) (Rey et al., 2017).

Genes suppressing *Ph1* and promoting homoeologous chromosome pairing have been reported in several wheat relatives. Examples are chromosome 5U of *Ae. umbellulata* Zhuk. (Riley et al., 1973), chromosomes 3E, 4E, and 5E of *Lophopyrum elongatum* (Host) Á. Löve (Dvořák, 1987), and chromosome 5M^g^ of *Ae. geniculata* Roth (Koo et al., 2016). Polymorphism for the suppression of *Ph1* was observed in *Ae. speltoides* and *Amblyopyrum muticum* (Boiss.) Eig (Dover and Riley, 1972; Dvořák, 1972; Kimber and Athwal, 1972). Using this polymorphism, major suppressors of *Ph1* were mapped on *Ae. speltoides* chromosome arms 3SL (*Su1-Ph1*) and 7SL (*Su2-Ph1*) (Dvorak et al., 2006b).

The first use of *Ph1* suppression to introgress a gene from a wheat relative into wheat employed hybridization of a wheat cytogenetic stock with *Ae. speltoides* (Riley et al., 1973). The presence of the *Ae. speltoides* genome induced recombination between the alien and wheat homoeologs. A logical extension of this idea is to introgress one of the *Ae. speltoides* suppressors into wheat. Such a wheat genetic stock would greatly simplify introgression of alien genes into wheat. Induction of recombination between homoeologous chromosomes would require nothing more than substituting the alien chromosome for a wheat homoeolog and crossing the substitution line with the *Ph1* suppressor line. The F_1_ would be doubly monosomic for the homoeologous chromosomes targeted for recombination and the expression of *Ph1* would be simultaneously suppressed by heterozygosity for the *Ph1* suppressor, provided that the suppressor is dominant. The first attempt to produce such a genetic stock resulted in the introgression of an *Ae. speltoides* suppressor named *Ph1*^I^ (Chen et al., 1994). The suppression of *Ph1* in this line is relatively weak (Chen et al., 1994). It is also not known what *Ae. speltoides* gene was introgressed and where *Ph1*^I^ is located in the wheat genome (Li et al., 2011). This situation has precluded the use of marker assisted selection (MAS) in genetic manipulations with this stock.

Another introgression of an *Ae. speltoides* suppressor into wheat occurred inadvertently as a by product of introgression of the leaf rust resistance gene *Lr66* located on chromosome 3S in *Ae. speltoides* (Marais et al., 2010). The introgressed gene could have been *Su1-Ph1*. The introgression was accompanied by sterility and has not been practically exploited.

Here, we report introgression of *Su1-Ph1* into the genetic background of hexaploid wheat (*T. aestivum*) cv. Chinese Spring (CS) and from there into the genetic background of tetraploid durum wheat (*T. turgidum* ssp. *durum*) cv. Langdon (LDN) utilizing MAS with *Xpsr1205-3S*. The marker is 0.2 to 0.4 cM distal to *Su1-Ph1*. MAS was aided by the development of an *Ae. speltoides*-specific assay for the *Xpsr1205-3S* haplotype (Dvorak et al., 2006b). The expression of the introgressed *Su1-Ph1* in the CS and LDN genetic backgrounds is evaluated and used to test the hypothesis that MAS for *Xpsr1205-3S* resulted in the introgression of *Su1-Ph1*. The locations of the *Su1-Ph1* relative to the *ZIP4-1* loci in the genomic sequences of chromosomes 3A and 3B of wild emmer (Avni et al., 2017) and that of chromosome 3D of *Ae. tauschii* (Luo et al., 2017) are used to test the hypothesis that *Su1-Ph1* is equivalent to *ZIP4-1*. A strategy for using the introgressed *Su1-Ph1* for introgression of alien genes in wheat is suggested.

## Materials and Methods

### Genetic Stocks

The initial material for introgression of *Su1-Ph1* into CS and LDN was *Ae. speltoides* F_4_ family #134. The family was derived from an F_2_ plant #134 in a population used for *Ph1* suppressor mapping (Dvorak et al., 2006b). The plant was homozygous for an active allele at *Su1-Ph1* and an inactive allele at *Su2-Ph1*. Family #134 inherited chromosome 3S with *Su1-Ph1* from *Ae. speltoides* accession PI 369609. The family was crossed with CS (accession DV148) to produce a CS × *Ae. speltoides* hybrid. To produce an octoploid amphiploid (2*n* = 8*x* = 56), the CS × *Ae. speltoides* #134 F_1_ hybrid was removed from its pot and the crown was immersed into 0.6% aqueous solution of colchicine overnight. The disomic substitution (DS) line of *L. elongatum* chromosome 3E for CS chromosome 3B, designated as DS3E(CS3B) (Tuleen and Hart, 1988) was employed in the initial stages of *Su1-Ph1* intorgression into CS. In addition, the DS lines of *Ae. searsii* chromosomes 1S^se^, 5S^se^, and 6S^se^ for their CS homoeologs, designated DS1S^se^(CS1A), DS1S^se^(CS1B), DS1S^se^(CS1D), DS5S^se^(CS5A), DS6S^se^(CS6A), and DS6S^se^(CS6D) (Friebe et al., 1995) were provided by N. E. Tuleen, Texas A&M University, College Station, TX, United States. The DS line of *L. elongatum* chromosome 1E in LDN, DS1E(LDN1A) and DS1E(LDN1B) (Jauhar and Peterson, 2011) were supplied by P. Jauhar, University of North Dakota, Fargo.

### Molecular Markers

A genome-specific PCR assay for *Xpsr1205-3S* (Dvorak et al., 2006b) was used in MAS for *Su1-Ph1* during backcrossing in the CS and LDN genetic backgrounds. Sequence information for wheat ESTs BE426080 and CD454867 and conserved primers of BE426080 (Forward- TGCACTTGCAAATCAAAAGC; Reverse- CGATCTTGCCACTCTTCTCC) and CD454867 (Forward- AGCTCCAGCAATCCTCTCAA; Reverse- GATGGTCGGCTATGCTCTTC) were obtained from the wheat single- nucleotide polymorphism (SNP) database^1^ (Akhunov et al., 2010). Using these conserved primers, amplicons were amplified from genomic DNAs of *Ae. searsii* accession TE10, DS5S^se^(CS5B), CS, and LDN and were treated with ExoSAP-IT (USB) and Sanger sequenced according to the manufacturers protocol (Applied Biosystems, Foster City, CA, United States). A homology search was then performed using BLAST at the bread wheat chromosome-based survey sequence^2^ site with these sequences. Alignments and comparisons of these sequences were used to discover SNPs at which the *Ae. searsii* nucleotide sequence differed from the wheat A-, B-, and D-genome nucleotide sequences. These polymorphisms were used to design *Ae. searsii* genome-specific primers. Their specificity was tested by PCR of DNAs of *Ae. searsii* accession TE10, DS5S^se^(CS5B), CS, and LDN. Two *Ae. searsii* chromosome 5S^se^-specific SNP markers, *BE426080-5S*^se^ (Forward- TTCTAGTAGAAGCTATTTCATGAGTAACTG; Reverse- CGATCTTGCCACTCTTCTCC) and *CD454867-5S*^se^ (Forward- AGCTCCAGCAATCCTCTCAA; Reverse- GAAAGGAGTTCAATGTGCTTCG) were developed and used for selection of plants with the 5S^se^ chromosome and for study of recombination between 5S^se^ and 5B.

Markers for studying recombination between LDN chromosome 1A and *L. elongatum* chromosome 1E were developed as follows. Markers were selected based upon their position on the *Ae. tauschii* SNP genetic map (Luo et al., 2013). Conserved primers were designed as described previously (Akhunov et al., 2010) and used to generate amplicons from *Ae. tauschii* accession AL8/78, *Ae. speltoides* line 134, *T. urartu* accession G1812, and *L. elongatum.* The amplicons were sequenced according to the protocol above. Amplicon sequences were aligned and differences between the sequences leveraged to design genome specific primers. E-genome specific primers (Supplementary Table S1) were tested for PCR amplification in genomic DNA of *L. elongatum*, *T. aestivum* cv. Chinese Spring, and DS1E(LDN1A) and DS1E(LDN1B).

The PCR reaction contained 1 unit of Taq DNA polymerase, 3 mM MgCl_2_, 50 pmol of forward and reverse primers, and 50 ng of template. The reaction conditions were as follows. DNA was denatured at 98°C for 5 min, which was followed by 40 cycles consisting of 96°C for 30 s, 30 s at annealing temperature, and extension at 72°C for 2 min. The 40 cycles were terminated by maintaining 72°C for 5 min. The amplicons were visualized by electrophoresis in a 2% agarose gel and stained with ethidium bromide.

### Cytological Observations

To verify that *Su1-Ph1* was introgressed into CS, BC_4_F_2_ plants heterozygous for *Xpsr1205-3S* were crossed with accessions G634, G637, G666, G1326, and G4984 of *Ae. peregrina* (Hack. in J. Fraser) Maire & Weiller (2*n* = 4*x* = 28) provided originally under the synonym *Ae. variabilis* by B. L. Johnson, University of California, Riverside, CA, United States. The presence of *Xpsr1205-3S* and the level of chromosome pairing was determined in each hybrid. *Ae. peregrina* accessions G1326 and G666 were also crossed with CS and the *ph1b* deletion mutant (Sears, 1977), respectively. To determine whether *Su1-Ph1* was active in the CS genetic background, B_5_F_2_ introgression plants heterozygous for *Xpsr1205-3S* were crossed with DS lines for *Ae. searsii* chromosomes 1S^se^ and 6S^se^ (Friebe et al., 1995) (provided by N. E. Tuleen, Texas A&M University, College Station, TX, United States). Hybrids were genotyped with *Xpsr1205-3S* and chromosome pairing was examined.

For chromosome pairing studies, immature spikes were collected from greenhouse-grown plants, fixed in freshly prepared Carnoy’s 6:3:1 (ethanol/acetic-acid/chloroform) fixative for 24 h at room temperature, and then stored in 70% ethanol. Squashes of pollen mother cells (PMCs) were stained with 2% acetocarmine. Meiotic chromosome pairing was scored in 20–30 meiocytes per plant. In the meiotic configurations, univalents, bivalents, trivalents, quadrivalents, and quinquevalents are indicated by I, II, III, IV, and V, respectively. The number of such configurations are indicated by an Arabic numeral preceding the symbol.

Mitotic chromosome spreads of tetraploid introgression lines were subjected to sequential fluorescence *in situ* hybridization (FISH) and genomic *in situ* hybridization (GISH). Chromosome spread preparation, probe labeling, and *in situ* hybridization were carried out according to the methods previously described by Li et al. (2014). In GISH procedures, genomic DNA of *Ae. speltoides* PI 369609 was labeled with ChromaTide Alexa Fluor-488-5-dUTP (Thermo Fisher Scientific, Waltham, MA, United States) as probe, and genomic DNA of LDN was used as blocking DNA. In FISH procedures, oligonucleotide probes Oligo-pTa535 and Oligo-pSc119.2 were used. Oligo-pTa535 and Oligo-pSc119.2 were reported to be equivalent to repetitive sequence probes in distinguishing wheat A-, B-, and D-genome chromosomes (Tang et al., 2014). Oligonucleotide probes labeled with 6-carboxyfluorescein (6-FAM) or 6- carboxytetramethylrhodamine (Tamra) at the 5′ end, were synthesized by Thermo Fisher Scientific. Photographs were captured with cellSens Standard 1.8 software (Olympus Corporation, Tokyo, Japan) on Olympus BX53 fluorescence microscope with a DP80 microscope digital camera (Olympus Corporation, Tokyo, Japan), and then processed with Adobe Photoshop CS 6.0 (Adobe Systems Incorporated, San Jose, CA, United States).

## Results

### Introgression of the *Ae. speltoides* Ph1 Suppressor *(Su1-Ph1)* into Hexaploid and Tetraploid Wheat

**FIGURE 1:**
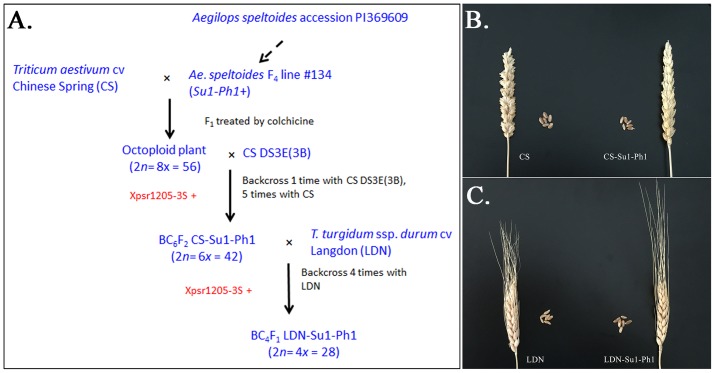
Introgression of *Su1-Ph1* into hexaploid and tetraploid wheat. **(A)** Breeding scheme culminating in the development of CS-Su1-Ph1 and LDN-Su1-Ph1. *Xpsr1205-3S* is an *Ae. speltoides*-specific marker tightly linked to *Su1-Ph1*. **(B)** Spike and seeds of *T. aestivum* cv. Chinese Spring (CS) and a CS-Su1-Ph1 introgression line. **(C)** Spike and seeds of *T. turgidum* ssp. *durum* cv. Langdon (LDN) and LDN-Su1-Ph1.

In our effort to introgress the entire *Ae. speltoides* chromosome 3S into wheat, the 56-chromosome amphiploid CS × *Ae. speltoides* was crossed with DS3E(CS3B) (**Figure 1A**). Ten F_1_ plants positive for the *Ae. speltoides Xpsr1205-3S* haplotype were obtained. The plants were male-sterile and were backcrossed once as females to DS3E(CS3B). The BC_1_F_1_ plants were again male sterile and were backcrossed as females to CS. Five male-sterile BC_2_F_1_ plants positive for *Xpsr1205-3S* were obtained and backcrossed once more as the female and male-fertile progeny was three times backcrossed as males to CS. Plants bearing *Xpsr1205-3S* were selected in each generation. The family of hexaploid introgression lines with *Xpsr1205-3S* in the CS genetic background was designated as CS-Su1-Ph1.

To introgress *Su1-Ph1* from CS-Su1-Ph1 into *T. turgidum* ssp. *durum* cv. LDN, a heterozygous BC_5_F_2_ plant (37^∗^95^∗^4^∗^14-8) was crossed with LDN and six *Xpsr1205-3S*-positive F_1_ plants were grown. The pentaploid hybrid was male sterile. It was backcrossed as the female parent to LDN four times, always selecting for the *Xpsr1205-3S* haplotype in progeny. Fertile plants positive for *Xpsr1205-3S* were ultimately obtained. They were designated as LDN-Su1-Ph1.

The CS-Su1-Ph1 lines were similar to CS in their morphology except for a pyramidal spike shape (**Figure 1B**). All CS-Su1-Ph1 plants, irrespective of the presence or absence of *Xpsr1205-3S*, had significantly lower seed set than CS but there was no difference in seed set between siblings with and without *Xpsr1205-3S* (**Table 1**) (one-way ANOVA, α = 0.05, LSD). Except for a single plant with a translocation, CS-Su1-Ph1 plants did not show multivalent chromosome pairing (**Table 2**).

**Table 1:** Mean number of seeds per spikelet in CS-Su1-Ph1 and LDN-Su1-Ph1 plants with and without *Xpsr1205-3S*, Chinese Spring and Langdon.

Family	Generation	No. plants	Chromosome number (2*n*)	*Xpsr1205-3S*	Seeds/spikelet
Chinese Spring	N/A	3	42	Absent	3.0
CS-Su1-Ph1	BC_5_F_3_	3	42 + 2 telosomes	Absent	2.1
CS-Su1-Ph1	BC_5_F_3_	3	42 + 2 telosomes	Present	2.2
CS-Su1-Ph1	BC_5_F_3_	7	42	Absent	2.1
CS-Su1-Ph1	BC_5_F_3_	7	42	Present	2.1
LDN-Su1-Ph1	BC_4_F_4-5_	12	28	Present	1.3
LDN-Su1-Ph1	BC_4_F_5_	1	28	Absent	2.4
Langdon	N/A	1	28	Absent	2.4


**Table 2:** Mean meiotic chromosome pairing in CS-*Su1-Ph1* and LDN-*Su1-Ph1* with the *Xpsr1205-3S* marker.

Stock	Generation	Pairing configuration^∗^				
		
		Cells	I	II	III	IV
CS-Su1-Ph1	BC_5_F_2_	6	0.2	19.0	0.2	0.8
	BC_5_F_4_	10	0.2	20.9	0.0	0.0
	BC_5_F_4_	11	0.6	20.7	0.0	0.0
	BC_5_F_4_	19	0.0	21.0	0.0	0.0
LDN-Su1-Ph1	BC_4_F_2_	19	0.4	13.4	0.0	0.2
	BC_4_F_2_	19	0.5	13.6	0.0	<0.1
	BC_4_F_4_	32	0.7	13.6	<0.1	0.0


*^∗^I = univalent; II = bivalent; III = trivalent; IV = quadrivalent.*

LDN-Su1-Ph1 lines were similar to LDN in spike shape, seed shape, and seed size. LDN-Su1-Ph1 seeds had darker pericarp than those of LDN (**Figure 1C**). LDN-Su1-Ph1 were less fertile than LDN (*P* = 0.001, *t*-test, *N* = 12 and 2, **Table 1**). All of the LDN-Su1-Ph1 plants showed a multivalent at MI in some PMCs (**Table 2**).

**FIGURE 2:**
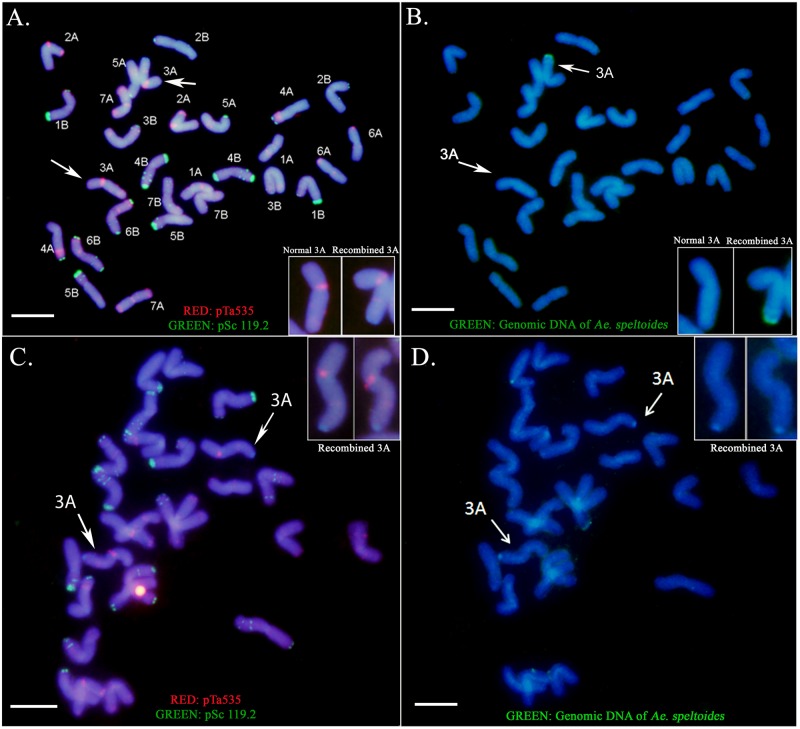
Sequential FISH and GISH karyotyping of LDN-Su1-Ph1. **(A,B)** The presence of a single recombined chromosome 3A with an *Ae. speltoides* segment replacing the distal portion of the long arm in LDN heterozygous for *Xpsr1205-3S*. **(C,D)** LDN homozygous for *Xpsr1205-3S* showing two recombined chromosomes 3A. The metaphase plate has only 27 chromosomes; one chromosome is outside of the field. **(A,C)** FISH identification of individual wheat chromosomes. **(B,D)** GISH identification of the recombined chromosomes. Scale bar = 10 μm.

To identify the wheat chromosome harboring the introgressed chromosome segment, sequential FISH and GISH were performed on LDN-Su1-Ph1. FISH with the oligo-pTa535 and oligo-pSc119.2 probes paints all wheat chromosomes and facilitates their identification (Tang et al., 2014). GISH with labeled genomic DNA of *Ae. speltoides* PI 369609 facilitated identification of the wheat chromosome harboring *Ae. speltoides* segments. Sequential FISH and GISH karyotyping of plants heterozygous for *Xpsr1205-3S* revealed the presence of a single recombined chromosome 3A with an *Ae. speltoides* segment replacing the distal portion of the long arm (**Figures 2A,B**). Sequential FISH and GISH karyotyping of plants homozygous for *Xpsr1205-3S*, showed two such chromosomes (**Figures 2C,D**). Thus, FISH and GISH karyotyping revealed that a single chromosome segment of *Ae. speltoides* was introgressed into LDN. The agreement between heterozygosity and homozygosity of the *Xpsr1205-3S* marker and the number of 3A chromosomes with the *Ae. speltoides* segments indicated that *Xpsr1205-3S* was located on that introgressed segment in LDN 3A.

### Chromosome Pairing in Hybrids Involving CS-Su1-Ph1

Previous studies suggested that the *Ae. peregrina* genome does not modify *Ph1* expression in wheat × *Ae. peregrina* hybrids (McGuire and Dvořák, 1982). If *Ph1* is active in the wheat parent, there would be little chromosome pairing in wheat × *Ae. peregrina* hybrids but if it is inactive, extensive pairing would take place.

Seven hybrids without *Xpsr1205-3S* were analyzed. One hybrid without *Xpsr1205-3S* differed from the remaining six by having an intermediate level of chromosome pairing. The remaining six hybrids without *Xpsr1205-3S* had an average of 2.7 chiasmata/PMC, which was comparable to the mean chiasma number in hybrids of CS × *Ae. peregrina* (**Table 3**). In contrast, the four hybrids having *Xpsr1205-3S* had an average of 16.6 chiasmata per PMC (*P* = 0.004, *t*-test, *N* = 11 and 4). We excluded one hybrid with an exceptionally low level of chromosome pairing, although it was positive for *Xpsr1205-3S* (**Table 3**). Two hybrids from the cross *ph1b* × *Ae. peregrina* had 18.6 chiasmata/PMC, which was still higher (*P* = 0.012, *t*-test, *N* = 3 and 2) than the mean chiasma frequency of hybrids with *Xpsr1205-3S*. Overall, *Ae. peregrina* hybrids seemed to confirm that MAS for *Xpsr1205-3S* resulted in introgression of *Su1-Ph1* into CS.

**Table 3:** Mean numbers of chiasmata per PMC in hybrids between allotetraploid *Aegilops peregrina* and Chinese Spring, CS *ph1b*, and CS-*Su1-Ph1* in the presence and absence of *Xpsr1205-3S.*

Accession	Female parent	*Xpsr1205-3S*	No. hybrids	Mean chiasmata/PMC^∗^
G1326	CS	-	3	3.7
G1326	CS-Su1-Ph1	-	1	3.7
G1326	CS-Su1-Ph1	+	1	16.8
G666	CS-Su1-Ph1	-	1	1.1
G666	CS-Su1-Ph1	-	1	12.7
G637	CS-Su1-Ph1	-	3	3.1
G637	CS-Su1-Ph1	+	1	1.6
G637	CS-Su1-Ph1	+	1	16.8
G4984	CS-Su1-Ph1	+	1	16.1
G634	CS-Su1-Ph1	-	1	1.5
G666	*ph1b*	-	2	18.6


*^∗^Mean number of chiasmata in hybrids CS-Su1-Ph1 × *Ae. peregrina* with and without *Xpsr1205-3S* significantly differed (*P* = 0.001, two-tailed *t*-test, *N* = 5 and 11).*

CS-Su1-Ph1 heterozygous for *Xpsr1205-3S* was crossed with *Ae. searsii* DS5S^se^(CS5A) and chromosome pairing was analyzed in progeny with and without *Xpsr1205-3S*. No PMC with all chromosomes paired was observed in a plant without *Xpsr1205-3S* (**Table 4**). Surprisingly, only 6.8% of PMCs showed complete pairing (a measure of pairing of the *Ae. searsii* 5S^se^ with the wheat homoeolog) in three progeny plants with *Xpsr1205-3S* (**Table 4**). This level of pairing between closely related homoeologous chromosomes was below what was expected for *Ph1* expression being suppressed.

**Table 4:** Percentages of PMCs with all 42 chromosomes paired indicating pairing between wheat and *Ae. searsii* homoeologous chromosomes in F_1_ progenies from crosses of *Ae. searsii* DS lines (parent 1) × CS-Su1-Ph1 or CS (parent 2).

Parent 1	Parent 2	*Xpsr1205-3S*	Plants	PMCs	% PMCs with complete chromosome pairing
DS1S^se^(CS1A)	CS-Su1-Ph1	-	1	28	3.6
	CS-Su1-Ph1	+	3	98	10.2
DS1S^se^(CS1B)	CS-Su1-Ph1	-	2	60	2.7
	CS-Su1-Ph1	+	1	32	6.3
DS1S^se^(CS1D)	CS	-	1	30	0.0
	CS-Su1-Ph1	-	1	11	0.0
	CS-Su1-Ph1	+	1	27	0.0
DS5S^se^(CS5A)	CS-Su1-Ph1	-	1	32	0.0
	CS-Su1-Ph1	+	3	87	6.8
DS6S^se^(CS6A)	CS-Su1-Ph1	-	1	27	0.0
	CS-Su1-Ph1	+	2	63	0.0
DS6S^se^(CS6D)	CS	-	1	22	0.0
	CS-Su1-Ph1	-	1	24	4.2
	CS-Su1-Ph1	+	4	125	0.0
Mean	CS	-			0.0
Mean^∗^	CS-Su1-Ph1	-			1.3
Mean^∗^	CS-Su1-Ph1	+			3.9


*^∗^The means do not statistically differ from each other (*P* = 0.16, two-tailed *t-*test, *N* = 6).*

To validate this result, CS-Su1-Ph1 heterozygous for *Xpsr1205-3S* was crossed with DS1S^se^(CS1A), DS1S^se^(CS1B), DS1S^se^(CS1D), DS6S^se^(CS6A), and DS6S^se^(CS6D). These DS lines were selected because chromosomes in these two homoeologous groups have not been reported to harbor genes affecting homoeologous chromosome pairing. DS lines for *Ae. searsii* chromosomes 1S^se^ and 6S^se^ were also crossed with CS as a control. PMCs that had complete chromosome pairing were rare irrespective of the presence or absence of *Xpsr1205-3S* (**Table 4**), suggesting that only minor or no suppression of *Ph1* took place in the presence of *Xpsr1205-3S.* This finding contradicted the conclusion made on the basis of chromosome pairing in hybrids involving *Ae. peregrina* and indicated that *Ae. peregrina* genome may not be entirely neutral with respect to suppression of *Ph1*.

### Chromosome Pairing and Recombination in the LDN Genetic Background

**FIGURE 3:**
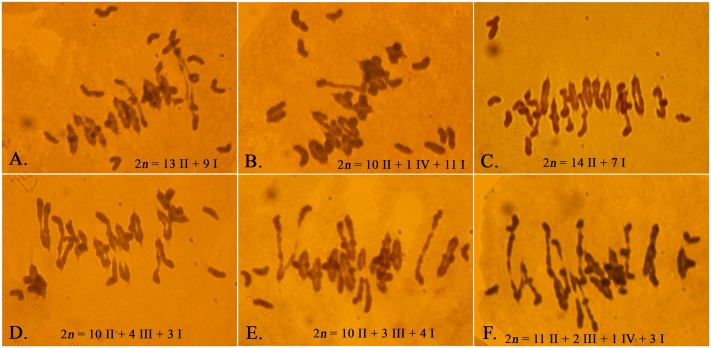
Meiotic chromosome pairing in F_1_ plants of LDN-Su1-Ph1 × Chinese Spring (2*n* = 35). **(A–C)** Pentaploid plants negative for *Xpsr1205-3S*. **(D–F)** Pentaploid plants positive for *Xpsr1205-3S*. Each PMC is from a different plant.

Because of concerns that the *Ae. peregrina* genome may be obscuring the true effects of the introgressed chromosome segment on the expression of *Ph1*, we used the CS genome in the assessment of the status of *Ph1* expression in the presence of the introgresed *Xpsr1205-3S.* LDN-Su1-Ph1 heterozygous for *Xpsr1205-3S* was crossed with CS and chromosome pairing was studied in progeny. If the chromosome segment harboring *Xpsr1205-3S* did not also harbor *Su1-Ph1*, the seven D-genome monosomes would not pair with their A- and B-genome homoeologs in the pentaploid hybrids (2*n* = 5*x* = 35) and 14II + 7I would be expected at MI in most PMCs. If, on the other hand, introgression of *Xpsr1205-3S* did result in the introgression of *Su1-Ph1* into LDN and *Su1-Ph1* suppressed *Ph1*, the D-genome chromosomes would pair with their A and B genome homoeologoues and trivalents accompanied by fewer than 7I would be expected in PMCs of the pentaploid hybrids with *Xpsr1205-3S*. In the four hybrids without *Xpsr1205-3S*, an average of 8.0 univalents and 13.4 bivalents were observed (**Table 5**). In one of these hybrids, an occasional trivalent and quadrivalent was also observed; none were observed in the other three hybrids. Chromosome pairing indicated that *Ph1* was active in all four hybrids. In the 11 hybrids with *Xpsr1205-3S*, the mean number of univalents per PMC was 5.8 (**Table 5**), which was significantly lower (*P* = 0.0002, two-tailed *t* = test with unequal variance) than the mean of 8.0 univalents per PMC in hybrids without *Xpsr1205-3S*. Concomitantly, the mean number of trivalents significantly increased to an average of 1.1 per PMC (*P <* 0.0001, two-tailed *t* = test with unequal variance). Up to four trivalents accompanied by three univalents were observed in a single PMC (**Figure 3**). These data were consistent with the hypothesis that *Ph1* was suppressed in the pentaploid hybrids with *Xpsr1205-3S* and indicated that introgression of *Xpsr1205-3S* to LDN was accompanied by introgression of an active *Su1-Ph1* allele.

**Table 5:** Meiotic pairing in pentaploid (2*n* = 35) progeny from the cross LDN-Su1-Ph1 × Chinese Spring.

Plant	*Xpsr1205-3S*	Pairing configuration^∗∗^					
		
		No. of cells	I	II	III	Range of III	IV
GH47583	+	21	6.7	12.9	0.8	0–2	<0.1
GH47584	+	20	5.9	13.1	0.9	0–3	<0.1
GH47585	+	27	5.4	13.0	1.1	0–3	<0.1
GH47588	+	25	5.6	13.2	1.0	0–3	0.0
GH47589	+	30	5.4	12.5	1.6	0–3	0.0
GH47590	+	24	5.7	12.7	1.3	0–3	0.0
GH47591	+	30	5.7	13.1	1.0	0–3	0.0
GH47586	-	32	7.7	13.7	0.0	0	0.0
GH47592	-	44	8.0	13.1	0.1	0–1	0.1
GH47593	-	32	8.7	13.2	0.0	0	0.0
GH47594	-	32	7.7	13.7	0.0	0	0.0
Mean^∗^	+		5.8a	12.9a	1.1a		<0.1a
Mean^∗^	-		8.0b	13.4a	<0.1b		<0.1a


*^∗^Means in columns followed by the same letter are not significantly different at the 5% significance level.*

*^∗∗^I = univalent; II = bivalent; III = trivalent; IV = quadrivalent.*

**FIGURE 4:**
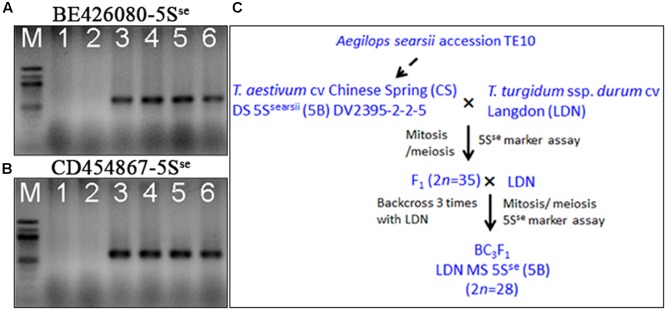
Development of a substitution line 5S^se^(5B)/5B in the LDN genetic background. **(A,B)** Two 5S^se^ chromosome specific SNP markers (*BE426080-5S*^se^ and *CD454867-5S*^se^) were developed. Lanes 1–6: LDN, CS, *Ae. searsii* TE10 (GH42258), *Ae. searsii* TE10 (GH42259), CS/*Ae. searsii* TE10 amphiploid (GH26353), CS/*Ae. searsii* TE10 amphiploid (GH26354). **(C)** Breeding scheme showing the development of the LDN double-monosomic substitution line 5S^se^(5B)/5B. Analyses performed in each step are in black.

Additional evidence confirming suppression of *Ph1* by *Su1-Ph1* in the LDN genetic background was provided by meiotic pairing of *Ae. searsii* chromosome 5S^se^ with LDN chromosome 5B. A double monosomic for *Ae. searsii* chromosome 5S^se^ and LDN chromosome 5B in the LDN genetic background (2*n* = 28) was developed as described in **Figure 4**. Plants from BC_1_ and BC_2_ generation were genotyped with *BE426080-5S*^se^ and *CD454867-5S*^se^ to select plants with chromosome 5S^se^ and crossed with LDN-Su1-Ph1 heterozygous for *Xpsr1205-3S.* In F_1_ plants with *Xpsr1205-3S*, the mean numbers of PMCs with complete chromosome pairing, indicating pairing of 5S^se^ with 5B, was 58.3%, and was significantly higher than mean of 7.9% of PMCs in F_1_ lacking *Xpsr1205-3S* (*P* < 0.01, two-tailed *t*-test with unequal variance) (**Table 6**).

**Table 6:** Meiotic chromosome pairing in the F_1_ plants from the crosses of two generations (BC_1_ and BC_2_) of monosomic substitution 5S^se^(LDN5B) × LDN-Su1-Ph1.

Plant	Generation	*Xpsr1205-3S*	5S^se^ marker	Pairing configuration^∗∗^					
				
				No. of cells	I	II	III	IV	% PMCs with complete chromosome pairing
GH36580	BC_1_	-	+	30	1.9	13.1	0.0	0.0	6.7
GH36585	BC_1_	-	+	29	1.8	13.1	0.0	0.0	10.3
GH45834	BC_2_	-	+	62	1.8	13.1	0.0	0.0	8.1
GH44117	BC_2_	-	+	32	1.9	13.1	0.0	0.0	6.3
GH36589	BC_1_	+	+	51	0.7	13.7	0.0	0.0	68.6
GH36586	BC_1_	+	+	40	0.7	13.7	0.0	0.0	65.0
GH36587	BC_1_	+	+	43	0.2	13.9	0.0	0.0	90.3
GH45830	BC_2_	+	+	35	1.4	13.2	<0.1	0.0	45.7
GH45831	BC_2_	+	+	25	1.0	13.4	<0.1	<0.1	56.0
GH45833	BC_2_	+	+	32	0.5	13.8	0.0	0.0	75.0
GH45835	BC_2_	+	+	50	1.0	13.5	0.0	0.0	56.0
GH45839	BC_2_	+	+	24	1.6	13.2	0.0	0.0	29.2
GH44153	BC_2_	+	+	70	1.5	13.3	0.0	0.0	38.6
Mean		-	+		1.9a	13.1a	0.0	0.0	7.9a
Mean		+	+		1.0b	13.5b	<0.1	<0.1	58.3b
*P*-value					<0.01	<0.01			<0.01


*^∗^Means followed by the same letter are not significantly different at the 5% significance level.*

*^∗∗^I = univalent; II = bivalent; III = trivalent; IV = quadrivalent.*

**FIGURE 5:**
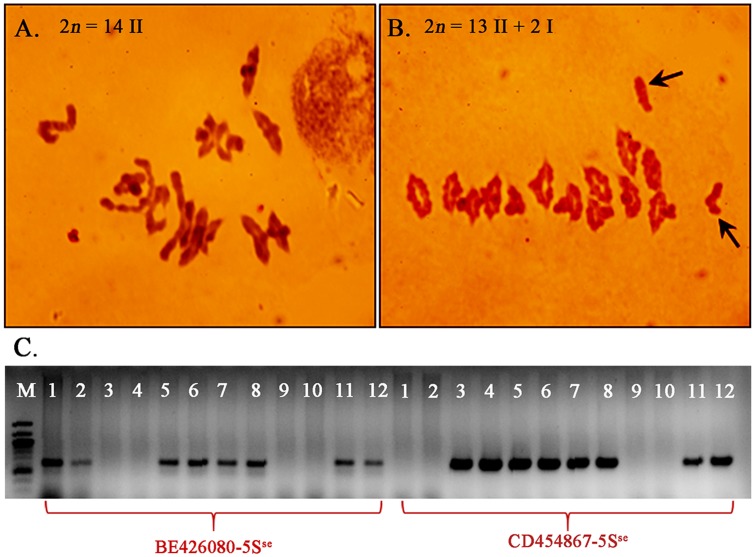
Meiotic pairing and recombination between chromosomes 5B and 5S^se^ induced by *Su1-Ph1*. Pollen mother cells showing pairing **(A)** and its absence **(B)** between chromosomes 5B and 5S^se^ in F_1_ progeny respectively with and without *Xpsr1205-3S* from the cross double monosomic 5S^se^(5B)/5B × LDN-Su1-Ph1. Arrows in **(B)** indicate the unpaired univalents 5B and 5S^se^. **(C)** Plants GH45780 and GH45804, with recombined chromosomes in the BE426080-CD454867 interval. From left, GH45780 (lanes 1–2), GH45804 (lanes 3–4), GH45837 (lanes 5–6), GH45793 (lanes 7–8), LDN (lanes 9–10), CS/*Ae. searsii* amphiploid (lanes 11–12).

The F_1_ plants were selfed and also backcrossed as females to LDN-Su1-Ph1 to study recombination. Genotypes at BE426080-5S^se^ and CD454867-5S^se^ were determined in 35 F_2_ and 28 BC_1_F_1_ progeny. The two markers are proximally located in the *Ph1* region of chromosome 5B and cosegregated at 160.6 cM on the genetic map from the cross of wild emmer (*T. turgidum* ssp. *dicoccoides*) × LDN (Jorgensen et al., 2017). Two recombined chromosomes, one in an F_2_ plant (GH45780) and the other in a BC_1_F_1_ plant (GH45804), were identified among 98 progeny chromosomes (**Figure 5**).

Chromosome pairing and recombination was also assessed between distantly related homoeologous chromosomes, the *L. elongatum* chromosome 1E and wheat chromosome 1A, in progeny from the cross LDN-Su1-Ph1 × DS1E(LDN1A). The *L. elongatum* chromosome 1E did not pair with wheat chromosome 1A (*N* = 64) in progeny without *Xpsr1205-3S* but it paired with it in 6.0% PMCs (*N* = 83) in progeny with *Xpsr1205-3S*. Based on the relationship 1% MI pairing = 0.5% recombination, the length of the 1E/1A linkage group (LG) was 3% recombination. Three recombined chromosomes were identified among 63 F_2_ progeny (**Table 7**). All three crossovers were validated by genotyping of 15 to 20 F_3_ progeny plants. The length of the 1E/1A LG based on recombination was 2.3%, which was comparable to 3% recombination based on MI pairing (*P* = 0.74, 2 × 2 contingency table). Two crossovers were located within the long arm and one was located within the short arm (**Table 7**). Thus, also this study confirmed that *Ph1* was suppressed in plants with *Xpsr1205-3S* in the LDN genetic background.

**Table 7:** Genotypes at seven SNP markers of the parental and recombined chromosomes in F_2_ plants of DS1E(LDN1A) × LDN-Su1-Ph1.

Plant	Short arm markers^∗^				Long arm markers^∗^		
		
	AT1D003^∗∗^	AT1D006	AT1D033	AT1D242	BE403420	AT1D659	BE446672
LDN	W	W	W	W	W	W	W
DS1E(LDN1A)	E	E	E	E	E	E	E
*L. elongatum*	E	E	E	E	E	E	E
GH21629	W	W	W	W	W	W	E
GH21638	E	E	W	W	W	W	W
GH21646	E	E	E	E	E	E	W


*^∗^W and E stand for 1A and *L. elognatum* alleles, respectively.*

*^∗∗^Markers are arranged as they are on the genetic map starting with the tip of the short arm to the left.*

## Discussion

### Introgression of *Su1-Ph1* into Wheat

Tight linkage of the *Xpsr1205-3S* marker to *Su1-Ph1* (Dvorak et al., 2006b) was exploited here in introgression of *Su1-Ph1* from *Ae. speltoides* into hexaploid wheat and from hexaploid wheat into tetraploid wheat. MAS was employed rather than selection for the meiotic pairing phenotype. Selection based only on meiotic phenotype would require a testcross with *Ae. peregrina*, or a similar tester, each backcross generation to ascertain that *Ph1* is suppressed. Moreover, it will be difficult to detect the presence of a suppressor allele in the CS background if the *Ph1* effect is not completely suppressed. Heterozygosity for translocations resulting from recombination between homoeologous chromosomes during backcrossing or from chromosome breakage and non-homologous end-joining due to the activity of gametocidal genes (Tsujimoto and Tsunewaki, 1984; Kota and Dvorak, 1988; Marais et al., 2010) could potentially lead to mistaking such multivalent pairing for homoeologous pairing.

The CS × *Ae. speltoides* hybrid treated with colchicine had dehiscent anthers and produced two octoploid seeds, indicating that it was male fertile but the two octoploid progeny plants were male sterile. They were therefore backcrossed as females with DS3E(CS3B) for two generations. Since *L. elognatum* chromosome 3E partially suppresses *Ph1* (Dvořák, 1987), it is therefore theoretically possible that the suppressor of *Ph1* introgressed in CS-Su1-Ph1 and LDN-Su1-Ph1 was the 3E suppressor rather than *Su1-Ph1*. This possibility is very unlikely for the following reasons. First, chromosome 3E has a weak suppressor, which is located in the short arm (Dvořák, 1987), whereas *Su1-Ph1* is a strong suppressor, which is located in the long arm. Second, the *Xpsr1205-3S* primers for PCR amplification were *Ae. speltoides* specific, and when DS3E(CS3B) was used as a template, no amplification was obtained. Therefore, using *Xpsr1205-3S* in introgression could not have introgressed a 3E segment in place of the targeted *Ae. speltoides* chromosome segment. Third, *in situ* hybridization showed that in plants with *Xpsr1205-3S*, the terminal segment of 3A was replaced by an *Ae. speltoides* chromosome segment. Finally, the LDN-Su1-Ph1 introgression lines were subjected to nine generations of backcrossing, first to CS and then to LDN. The probability that a recombined chromosome bearing the 3E suppressor was present in the LDN-Su1-Ph1 lines was <0.002.

Five approaches were employed to test the hypothesis that *Su1-Ph1* was introgressed along with introgression of *Xpsr1205-3S* with the following results. (1) D-genome chromosomes frequently paired in trivalents with their A- and B-genome homoeologs in the pentaploid LDN-Su1-Ph1 × CS hybrids that acquired *Xpsr1205-3S* but not in those that did not acquire *Xpsr1205-3S*. (2) Chromosome 5S^se^ paired with LDN chromosome 5B in 58.3% of PMCs if *Xpsr1205-3S* was present but only in a few percent of PMCs if *Xpsr1205-3S* was absent. Recombined 5S^se^/5B chromosomes were recovered in progeny of plants with *Xpsr1205-3S.* (3) *L. elongatum* chromosome 1E paired in the LDN genetic background with chromosome 1A if *Xpsr1205-3S* was present but did not pair with it if *Xpsr1205-3S* was absent. Recombined 1E/1A chromosomes were recovered in progeny. (4) A mean of 16.6 chiasmata per PMC was observed in F_1_ hybrids CS-Su1-Ph1 × *Ae. peregrina* if *Xpsr1205-3S* was present but only 2.7 chiasmata/PMC were observed if *Xpsr1205-3S* was absent. The last approach (5) provided evidence contradictory to that provided by approaches (1) to (4). Single *Ae. searsii* chromosomes 1S^se^, 5S^se^, and 6S^se^ paired with wheat homoeologs in only a few percent of PMCs in progenies involving CS-Su1-Ph1 in the presence of *Xpsr1205-3S*. Thus, the presence of the introgressed *Xpsr1205-3S* consistently resulted in suppression of *Ph1* in the LDN genetic background but the results were inconsistent in the CS genetic background.

Sequential FISH and GISH showed that LDN-Su1-Ph1 plants with *Xpsr1205-3S* harbored an *Ae. speltoides* chromosome segment replacing a distal portion of the long arm of chromosome 3A. The correct orientation of the segment in the arm suggests that the translocation originated by recombination between LDN chromosome 3A and *Ae. speltoides* chromosome 3S. We name the chromosome as T(3AL;3SL)Dv1.

The LDN T(3AL;3SL)Dv1 chromosome was introgressed into LDN from CS-Su1-Ph1. If the T(3AL;3SL)Dv1 chromosome harbored *Su1-Ph1* in LDN, the chromosome must have also harbored the *Su1-Ph1* in CS-Su1-Ph1. To account for these puzzling contradictions, we hypothesize that *Su1-Ph1* normally interacts with at least one other gene in *Ae. speltoides* that was lost during introgression. We further hypothesize that this complementary gene is absent in the CS genome but present in the LDN and *Ae. peregrina* genomes.

Evidence for the presence of the complementary gene in LDN was provided by extensive homoeologous chromosome pairing in the LDN-Su1-Ph1 × CS pentaploid hybrids with *Xpsr1205-3S*. The fact that no homoeologous chromosome pairing took place in the same hybrids devoid of *Xpsr1205-3S* suggested that the LDN gene affects chromosome pairing only when complemented by *Su1-Ph1*.

*Aegilops peregrina* (genomes UUS^v^S^v^) and tetraploid wheat (genomes AABB) each have one genome pair closely related to the S genome of *Ae. speltoides* (Dvorak, 1998; Badaeva et al., 2004) and each could have possessed this complementary gene we postulate to exist in *Ae. speltoides. Ae. peregrina* accession G637 may be polymorphic for the gene, as the hybrid with *Xpsr1205-3S* but with low pairing may suggest.

Chromosome pairing in the LDN-Su1-Ph1 and CS-Su1-Ph1 introgression lines is consistent with the hypothesis that the complementary gene(s) is present in LDN but not in CS. Occasional multivalents from heterogenetic chromosome pairing were observed in all LDN-Su1-Ph1 plants. In contrast, no multivalents were observed in the CS-Su1-Ph1 plants.

Since tetraploid and hexaploid wheat share the A and B genomes, and since there has been extensive gene flow between tetraploid and hexaploid wheat (Dvorak et al., 2006a; Akhunov et al., 2010), it is very likely that hexaploid wheat is polymorphic for the complementary gene. A search for the gene in *T. aestivum* will require the development of a complementation assay that would indicate the presence of homoeologous chromosome pairing in hybrids involving *Xpsr1205-3S*.

### Molecular Nature of *Su1-Ph1* and Its Relationship to *ZIP4-1* and *Zip4-B2*

**FIGURE 6:**
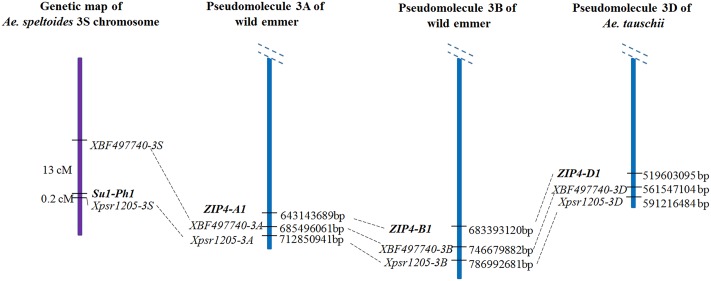
Molecular nature of *Su1-Ph1* and relationship to *ZIP4-1. Su1-Ph1* was flanked by *Xpsr1205-3S* (0.2 cM) and EST locus *XBF497740* (13 cM) (Dvorak et al., 2006b). The order of the three loci on the 3A and 3B wild emmer pseudomolecules (Avni et al., 2017) and the 3D *Ae. tauschii* pseudomolecule (Luo et al., 2017) is as shown. *ZIP4*-1 is about 30 Mb proximal to the *XBF497740* marker and about 70 Mb proximal to *Xpsr1205*, which indicates that it is very unlikely that *ZIP4-1* and *Su1-Ph1* are equivalent.

The *Su1-Ph1* locus and *ZIP4-1* gene, the likely source of the *Ph1* gene (Rey et al., 2017), are both located on the long arm of chromosome 3A. Does the spatial and functional relationship of *Su1-Ph1* and *ZIP4-1* indicate equivalence of the two loci? The locations of these loci on the reference genome sequences of wild emmer (Avni et al., 2017) and *Ae. tauschii* (Luo et al., 2017) suggest that they are not equivalent. *Su1-Ph1* was mapped between *Xpsr1205-3S* and EST locus *XBF497740* on two independent maps. *Su1-Ph1* and *XBF497740* were 0.2 and 13 cM proximal to *Xpsr1205-3S*, respectively (Dvorak et al., 2006b). Orthologous *Xpsr1205* loci are on the 3A, 3B, and 3D pseudomolecules at 712,850,941, 786,992,681, and 591,216,484 bp, respectively (**Figure 6**). The orthologous *XBF497740* loci are about 30 Mb proximal to *Xpsr1205* in each pseudomolecule (**Figure 6**) but the *ZIP4-A1* and *ZIP4-B1* and *ZIP4-D1* loci, which are at collinear locations on pseudomolecules 3A, 3B and 3D, are about 30 Mb further proximal to the *XBF497740* marker and about 70 Mb proximal to *Xpsr1205*. It is therefore very unlikely that *ZIP4-1* and *Su1-Ph1* are equivalent. Whether *ZIP4-B1* is actually the complementary locus detected in LDN or whether *Su1-Ph1* regulates the expression of *ZIP4-B1* and *ZIP4-B2* must be investigated.

Comparison of chromosome pairing in hybrids of frameshift mutants of *ZIP4-B2* (Cad1691 and Cad0348) × *Ae. peregrina* (Rey et al., 2017) with the hybrids of CS-Su1-Ph1 × *Ae. peregrina* suggests that *Ph1* expression was not entirely abolished by the Cad1691 and Cad0348 mutations. The hybrids had an average of 12.2 chiasmata/PMC (Rey et al., 2017) whereas those involving CS-Su1-Ph1 had an average of 16.6 chiasmata/PMC (*P* < 0.001, *t*-test, *N* = 3 and 2). Both sets of hybrids had lower chiasma frequency than the hybrids involving the *ph1b* deletion mutation (18.6 chiasmata/PMC). The average number of chiasmata in hybrids involving the *ZIP4-B2* mutants was similar to the exceptional hybrid involving CS-Su1-Ph1 × *Ae. peregrina* accession G666, which showed an intermediate level of chromosome pairing (12.7 chiasmata/PMC). The mutants of *ZIP4-B2* did not show multivalents at MI in meiosis (Rey et al., 2017), which is consistent with partial activity of *Ph1*. Evidence that *ZIP4-B2* is equivalent to *Ph1* hinges on chromosome pairing in the hybrids with *Ae. peregrina*. As we have learned, the *Ae. peregrina* genome can obscure the actual effect of genes affecting *Ph1*, and it would be prudent to provide additional evidence that *Ph1*expression was abolished in Cad1691 and Cad0348 *ZIP4-B2* mutants.

### Practical Utility of the Introgressed *Su1-Ph1*

Since the presence of heterozygous *Xpsr1205-3S* in the LDN-Su1-Ph1 × CS F_1_ hybrids elicited homoeologous chromosome pairing, *Su1-Ph1* must be dominant. The dominant epistasis of *Su1-Ph1* over *Ph1* makes it a very flexible means of inducing recombination between homoeologs. To achieve recombination between an alien chromosome and a wheat homoeolog, the alien chromosome should be substituted for a wheat homoeolog and the substitution line should be crossed with an introgression line harboring the T(3AL;3SL)Dv1 chromosome. The introgression line supplies both the *Ph1* suppressor and the wheat homoeologs for recombination with the alien chromosome. This strategy was illustrated here by targeting recombination between homoeologous chromosomes 5S^se^ with 5B and 1E with 1A.

There are however two caveats. One is that *Su1-Ph1* will suppress *Ph1* only if one of the parents contributes the complementary gene to F_1_ progeny. This requirement is satisfied by the LDN-Su1-Ph1 introgression lines but not by the CS-Su1-Ph1 introgression lines. The alien chromosome substitution lines should therefore be in the LDN genetic background, as was done here to achieve recombination of 5S^se^ with 5B and 1E with 1A. It is also possible to use hexaploid alien chromosome substitutions and rely on the induction of recombination between the homoeologs in the pentaploid hybrids, but that strategy has not been tested.

The other caveat is that *Su1-Ph1* does not suppress *Ph1* completely. The chiasma frequency in CS-Su1-Ph1 × *Ae. peregrina* F_1_ hybrids was significantly lower than the average chiasma frequency in the *ph1b × Ae. peregrina* F_1_ hybrids. Therefore, *Su1-Ph1* will be the most effective in the manipulation of closely related homoeologous chromosomes, although, as illustrated here, even more distantly related chromosomes, such as *L. elongatum* and wheat, could be recombined.

## Author Contributions

JD, HL, and AD conceived and designed the experiments. HL, KD, and JD performed the experimental work. JD, HL, and KD analyzed the data. JD, HL, KD, M-CL, WJ, and AD discussed the findings and interpreted the results. JD and HL wrote the first draft of the paper. All authors have read and approved the final draft.

## Conflict of Interest Statement

The authors declare that the research was conducted in the absence of any commercial or financial relationships that could be construed as a potential conflict of interest.

## Footnotes

1 https://wheat.pw.usda.gov/SNP/new/index.shtml

2 https://urgi.versailles.inra.fr/blast/blast.php
